# IL-6 and IL-10 Are Associated With Gram-Negative and Gram-Positive Bacteria Infection in Lymphoma

**DOI:** 10.3389/fimmu.2022.856039

**Published:** 2022-04-01

**Authors:** Qiuhua Zhu, Huan Li, Shanshan Zheng, Bin Wang, Mingjie Li, Wenbin Zeng, Lanlan Zhou, Zebing Guan, Hong Wang, Yanan Liu, Yanmin Gao, Shiqiu Qiu, Chaolun Chen, Shimei Yang, Yuemei Yuan, Hanling Zhang, Guanqiao Ruan, Xueyi Pan

**Affiliations:** ^1^Department of Hematology, The First Affiliated Hospital of Guangdong Pharmaceutical University, Guangzhou, China; ^2^Department of Organ Transplantation, The Third Affiliated Hospital of Guangzhou Medical University, Guangzhou, China

**Keywords:** Th1/Th2 cytokines, IL-6, IL-10, lymphoma, infection, bacteria

## Abstract

To investigate the Th1/Th2 cytokine profile in patients with lymphoma during the myelosuppression stage of infection. 52 patients with gram-negative bacterial infection (G- group), 49 patients with gram-positive bacterial infection (G+ group), 51 uninfected patients with lymphoma (uninfected group) and 20 healthy controls (healthy group) were enrolled in this study. We evaluated the quantification of Th1/Th2 cytokines with flow cytometry bead assay (CBA) in the sera to explore a rapid diagnostic method to determine the type of infection and anti-infective effect. The levels of procalcitonin (PCT) were also detected simultaneously. The four groups did not differ with regard to IL-2 and IL-4 (P>0.05). The IFN-γ and TNF-α levels of patients with lymphoma were higher than those of healthy controls (P<0.05). There was significantly upregulated IL-6 and IL-10 expression in the G- group (P<0.001). A similar trend was reflected in the IL-6 of the G+ group, which was significantly increased (P<0.001). However, no significant upregulation was observed for IL-10 in the G+ group. According to the different degrees of increased IL-6 and IL-10 levels, We proposed to use the G- Bacterial Infection Cytokine Profile (G- BICP) and the G+ Bacterial Infection Cytokine Profile (G+ BICP) for the first time to differentiate between Gram-negative and Gram-positive (G-/G+) bacterial infection in adults with lymphoma in the myelosuppression stage after chemotherapy. The IL-6, IL-10 and PCT in the G- group and the IL-6, PCT in the G+ group were significantly decreased at day 4 and day 8 compared with those at day 1. IL-6 and IL-10 are closely associated with the severity and treatment efficacy in adults with lymphomas who develop infections after chemotherapy and can help distinguish between G- and G+ bacterial infections at an early stage.

## Introduction

Lymphoma is a malignant tumour originating in the lymph nodes and/or extranodal lymphoid tissues. The main symptoms include painless lymphadenopathy, hepatosplenomegaly, accompanying whole-body and multiorgan reactions such as fever, wasting, drenching night sweats and itching (1). Myelosuppression often occurs in lymphoma patients who are treated with chemotherapy. During the myelosuppression stage, especially in the case of granulocytosis, patients have lower immunity and are prone to infection (2–4). Infection not only increases medical costs but also causes chemotherapy dose reductions, dose delays, or even treatment discontinuation that may result in prolonged hospitalization and reduced overall survival. Timely and effective anti-infective treatment is very important, and timely identification of infectious pathogens is critical for anti-infective efficacy. At present, the exact method for identifying pathogens is the cultivation of pathogenic bacteria (including blood culture, sputum culture and urine culture). However, it takes a certain length of time for the results of the cultivation of pathogenic bacteria, one or two days at least and as long as three or more days. Although elevated PCT and CRP usually indicate infection, G-/G+ infection cannot be distinguished. Therefore, it is necessary to find a new and faster method to identify G-/G+ infection.

Helper T (T helper, Th) cells have an important regulatory effect on the body’s immune system (5). Th can be divided into the TH1 subgroup and the TH2 subgroup under the stimulation of different antigens (6–8). Th1 cells mainly secrete IL-2 and IFN-γ (9, 10), which are involved in regulating cellular immunity, assisting the differentiation of cytotoxic T cells and participating in delayed hypersensitivity reactions. Th2 cells mainly secrete IL-4, IL-5, IL-6 and IL-10 (9–13), and their main function is to stimulate the proliferation of B cells and produce antibodies, which are related to humoral immunity and involved in the formation of immunosuppression, immune tolerance and rapid type hypersensitivity reactions. In recent years, it has been reported that Th1/Th2 cytokine changes play an important role in many infectious diseases (14, 15). Tang et al. used IL-6/IL-10 cytokines to identify G-/G+ bacteria in children with haematological diseases (15). However, there are few studies on the changes in THI/TH2 cytokines in adults with lymphomas who develop infection after chemotherapy. This study aims to quickly identify bacterial types through IL-6 and IL-10, select appropriate antibiotics in a timely manner, and understand the efficacy of anti-infection treatment through the trends of IL-6 and IL-10.

## Materials and Methods

### Select Objects

This retrospective study was approved by the Ethics Committee of the First Affiliated Hospital of Guangdong Pharmaceutical University. From January 2018 to September 2021, 52 lymphoma patients with gram-negative bacterial infection and 49 patients with gram-positive bacterial infection after chemotherapy treatment in the Haematology Department of our institution were enrolled. which were defined as G- group and G+ group, respectively. 51 patients with lymphoma and no infection after chemotherapy were enrolled as the uninfected group, and 20 healthy adults were enrolled as healthy controls. Inclusion criteria were as follows: Patients with lymphoma after chemotherapy who had granulocytopenia; cultures of pathogenic bacteria (blood, sputum, or midstream urine) detected gram-negative or gram-positive bacteria. Exclusion criteria were as follows: younger than 18 years old; a diagnosis of other malignant tumours or haemophagocytic syndrome; culture results that were not single strain or specimen contamination; a diagnosis of rheumatic connective tissue disease; pregnancy and perinatal period. The need for informed consent was waived by the committee.

### Specimen Collection and Detection

Culture of pathogenic bacteria (including blood culture, sputum culture and urine culture) was performed on lymphoma patients after chemotherapy when fever first appeared during the myelosuppression stage. Peripheral venous blood was collected early in the morning on an empty stomach from patients in the G- group and G+ group, healthy group and uninfected group. The concentrations of IL-2, IL-4, IL-6, IL-10, tumour necrosis factor-α (TNF-α) and interferon-γ (INF-γ) in the sera were quantitatively determined by the CBA HumanTh1/Th2 Cytokine Kit II (BD Biosciences, San Jose, CA) as previously described in the literature (5, 15). The normal ranges of IL-6 and IL-10 were 1.18-5.30 pg/ml and 0.19-4.91 pg/ml, respectively. The levels of IL-6, IL-10 and PCT in the G- and G+ groups were further detected on day 1 (the first day of treatment), day 4 and day 8.

### Statistical Analysis

All statistical analyses were performed using SPSS 19.0 (IBM Corp., Armonk, NY). Continuous variables were summarized by the mean, standard deviation (SD), median, and range, while categorical variables were summarized by number and frequencies (%). Categorical variables were compared using chi-square tests. Comparisons of continuous variables among three or more groups were carried out using one-way analysis of variance (ANOVA) followed by a nonparametric test when data did not follow a normal distribution or homogeneity of variance. The diagnostic value of each indicator was appraised employing receiver operating characteristic (ROC) curves. The 95% confidence interval was utilized to calculate the area under the curve (AUC), and P values <0.05 (two-sided) were considered statistically significant.

## Results

### Baseline Clinical Characteristics

**Table 1** shows the clinical features of the four groups. The enrolled numbers of the G- group, the G+ group, the uninfected group and the healthy group were 52, 49, 51 and 20, respectively. The four groups did not differ with regard to gender and age (P>0.05, **Table 1**). No significant differences were observed in the leukocyte count, neutrophil count or type of lymphoma among the G- group, G+ group and uninfected group. (P>0.05, **Table 1**). Infective microorganisms mainly included Escherichia coli (25, 48.1%), Klebsiella pneumoniae (11, 21.1%), Pseudomonas aeruginosa (7, 13.5%) and Stenotrophomonas maltophilia (4, 7.7%) in the G- group, and the G+ group mainly included Enterococcus faecalis (15, 30.6%), Staphylococcus epidermidis (12, 24.5%), Staphylococcus aureus (11, 22.4%) and Staphylococcus hominis (4, 8.2%) (**Table 2**).

**Table 1:** Baseline characteristics of the four groups.

	G-	G+	Uninfected	Healthy	P value
	(N=51)	(N=50)	(N=51)	(N=20)	
Male, n (%)	27 (51.9)	26 (53.1)	26(51.0)	11(55.0)	0.992
Age (years),					
Mean ± SD	44.3 ± 17.0	44.7 ± 16.0	44.9 ± 15.4	44.6 ± 14.3	0.990
Range	19-86	19-80	20-79	19-70	
Leukocyte count (×109/L)					0.801^a^
Mean ± SD	1.6 ± 0.7	1.6 ± 0.6	1.7 ± 0.7	/	
Range	0.54-3.41	0.67-3.48	0.63-3.63		
Neutrophil count (×109/L)					0.393^b^
Mean ± SD	0.6 ± 0.3	0.7 ± 0.3	0.7 ± 0.3	/	
Range	0.21-1.35	0.26-1.41	0.32-1.51		
Type of lymphoma, n (%)					0.996^c^
DLBCL	20 (38.5)	19 (38.8)	21(41.2)	/	
T or B lymphoblastic lymphoma	7 (13.5)	6 (12.2)	8(15.7)	/	
NK/T-cell lymphoma	9 (17.3)	7 (14.3)	9(17.6)	/	
PTCL	9(17.3)	10 (20.4)	7(13.7)	/	
Burkitt lymphoma	7 (13.5)	7 (14.3)	6(11.8)	/	

DLBCL, diffuse large B-cell lymphoma; NK/T-cell lymphoma, natural killer/T-cell lymphoma; PTCL, peripheral T-cell lymphoma.

^a^P value compares leukocyte count among the G- group, the G+ group and the uninfected group.

^b^P value compares neutrophil count among the G- group, the G+ group and the uninfected group.

^c^P value compares type of lymphoma among the G- group, the G+ group and the uninfected group.

**Table 2:** Classification of bacterial infections in G- and G+ groups.

G- group	N(%)	G+ group	N(%)
Escherichia coli	25(48.1)	Enterococcus faecalis	15(30.6)
Klebsiella pneumoniae	11(21.1)	Staphylococcus epidermidis	12(24.5)
Pseudomonas aeruginosa	7(13.5)	Staphylococcus aureus	11(22.4)
Stenotrophomonas maltophilia	4(7.7)	Staphylococcus hominis	4(8.2)
Acinetobacter baumannii	3(5.8)	Staphylococcus haemolyticus	3(6.1)
Aeromonas temperate	1(1.9)	Unilateral Staphylococcus cephalus	1(2.0)
Salmonella dubos	1(1.9)	Bacillus	2(4.1)
		Rhodococcus equi	1(2.0)
Total	52(100)	Total	49(100)

### Th1/Th2 Cytokines Among the Four Groups

The four groups did not differ with regard to IL-2 and IL-4 (P=0.981, P=0.522, respectively, **Table 3**). There were no significant differences in INF-γ and TNF-α among the G- group, the G+ group and the uninfected group (P=1.000, **Table 3**). The INF-γ and TNF-α levels of the G- group, the G+ group and the uninfected group were higher than those of the healthy group (P<0.05, **Table 3**). The IL-6 and IL-10 levels of the G- group were 386.1(26.8-14785.5) pg/ml and 123.8 (2.6-9327.0) pg/ml, respectively, which were significantly higher than those of the uninfected group at 5.8 (0.9-67.6) pg/ml and 2.6 (0.2-26.8) pg/ml and the healthy group at 1.1 (0.3-7.7) and 1.0 (0.6-9.9) pg/ml (P<0.001, **Table 3**). The IL-6 level of the G+ group was 501.0 (19.3-2134.7) pg/ml, which was significantly higher than that of the uninfected group 5.8 (0.9-67.6) pg/ml and the healthy group (1.1 (0.3-7.7) pg/ml) (P<0.001, **Table 3**). However, no significant difference was observed for IL-10 between the G+ group 9.4 (0.5-64.2) pg/ml and the uninfected group 2.6 (0.2-26.8) pg/ml (P=0.084, **Table 3**).

**Table 3:** Th1/Th2 cytokines among the four groups.

Groups	n	IL-2 (pg/ml)	IL-4 (pg/ml)	IL-6 (pg/ml)	IL-10 (pg/ml)	INF-γ (pg/ml)	TNF-α (pg/ml)
Healthy(A)	20	0.6(0.3-6.2)	0.6(0.2-4.3)	1.1 (0.3-7.7)	1.0 (0.6-9.9)	0.7 (0.6-0.9)	0.6 (0.1-1.5)
Uninfected (B)	51	0.9 (0.1-5.1)	0.7 (0.1-4.9)	5.8(0.9-67.6)	2.6(0.2-26.8)	3.8 (0.2-129.7)	1.2 (0.1-173.1)
G-Group(C)	51	0.9 (0.1-5.6)	1.0 (0.1-6.7)	386.1(26.8-14785.4)	123.8(2.6-9327.0)	4.0 (0.2-136.9)	1.4 (0.1-181.6)
G+ Group(D)	50	0.8 (0.1-6.0)	0.8 (0.1-4.8)	501.0 (19.3-2134.7)	9.4 (0.5-64.2)	2.8 (0.1-134.5)	1.3 (0.1-130.3)
P0		0.981	0.522	0.000	0.000	0.000	0.01
P1(A vs. B)				0.0483	0.364	0.000	0.026
P2(A vs. C)				0.000	0.000	0.000	0.012
P3(A vs. D)				0.000	0.001	0.000	0.012
P4(B vs. C)				0.000	0.000	1.000	1.000
P5(B vs. D)				0.000	0.084	1.000	1.000
P6(C vs. D)				1.000	0.000	1.000	1.000

P0: P value compares cytokine levels among the four groups.

**Figure 1:**
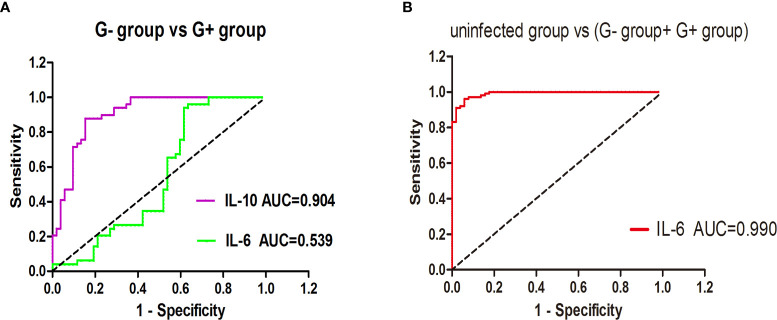
The ability of IL-6/IL-10 to distinguish the G- group and G+ group, uninfected group and infected group. **(A)** shows the ROC curve for distinguishing G- bacterial infection and G+ bacterial infection; **(B)** shows the ROC curve for distinguishing the infected group and uninfected group.

**Figure 2:**
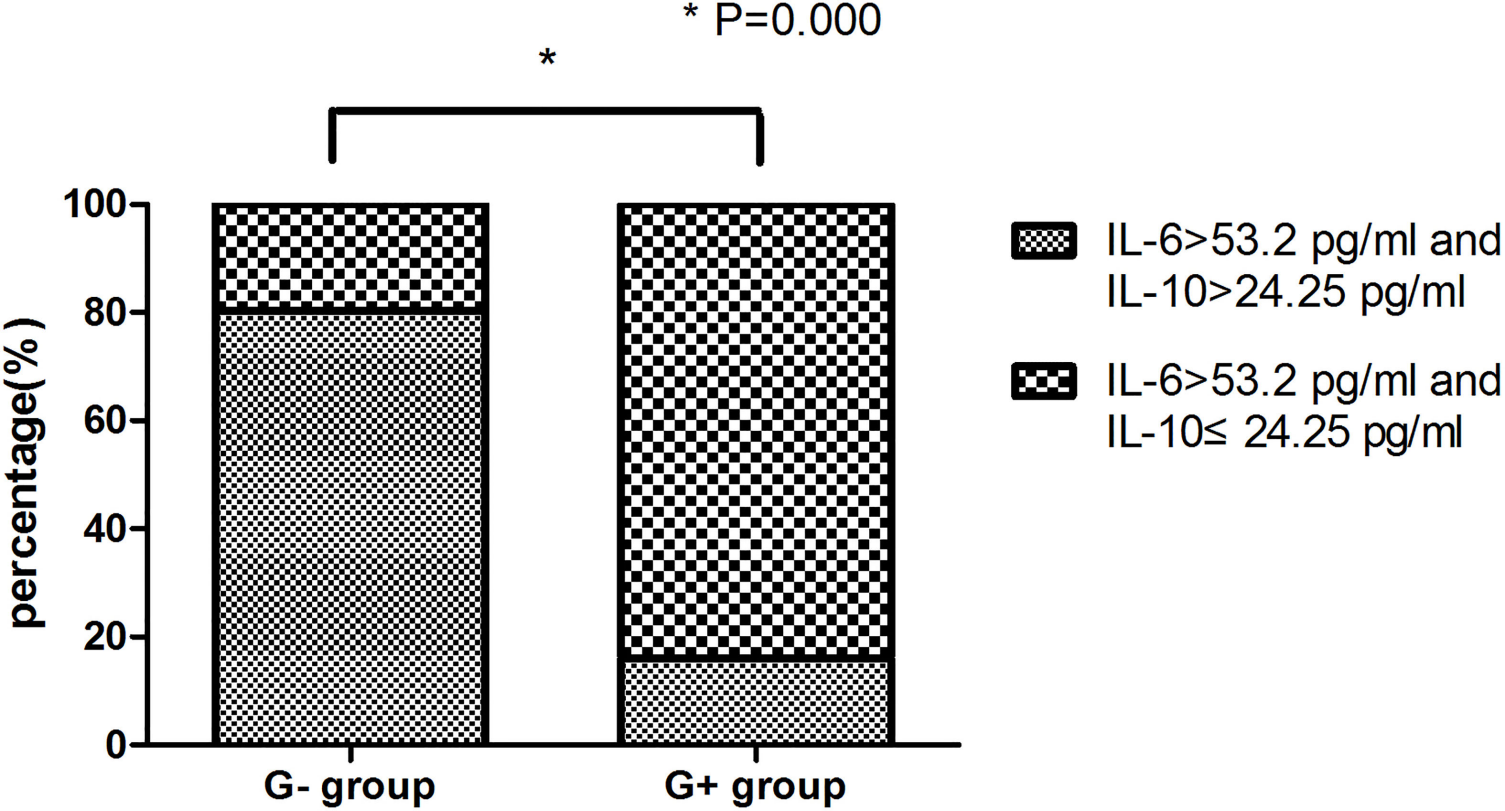
Comparison between the G- group and G+ group.

**Figure 3:**
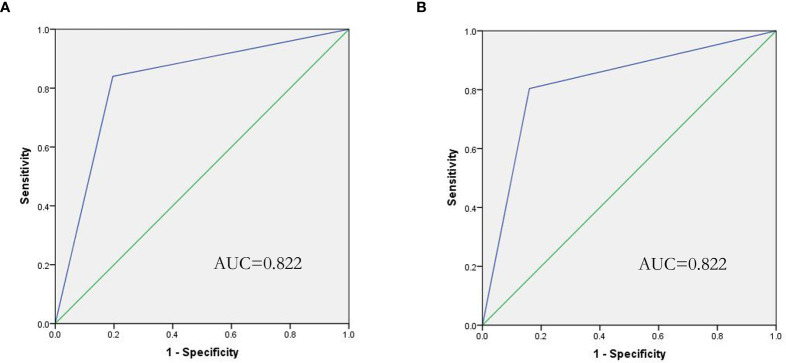
ROC curve of the IL-6/IL-10 cytokine profile for the prediction of G-/G+ bacterial infection. **(A)** Shows that IL-6/IL-10 predicts G- bacterial infection (AUC=0.822), and **(B)** shows that IL-6/IL-10 predicts G+ bacterial infection (AUC=0.822).

In this study, a significant increase in IL-6 levels was found in the presence of G+ and G- bacterial infections. To further distinguish between G- and G+ bacterial infections, we generated ROC curves for the G- group and G+ group (**Figure 1A**). We found that IL-10 could distinguish patients in the G- and G+ groups (**Figure 1A**, AUC = 0.904, P=0.000). Considering that the cut-off value of IL-10 is 24.25 pg/mL, its sensitivity and specificity are 87.8% and 84.6%, respectively. However, there was no significant difference in the ROC value of IL-6 between the G- and the G+ groups (AUC=0.539, P=0.501). We further performed ROC curve analysis in the infected group (G- and G+ groups) and the noninfected group (**Figure 1B**). We found that IL-6 could distinguish the infected group from the uninfected group (**Figure 1B**, AUC = 0.990, P=0.000). The cut-off value of IL-6 was 53.20 pg/mL, and the sensitivity and specificity were 96.0% and 94.1%, respectively. A single IL-6 level is not enough to predict the type of bacterial infection in all populations. Therefore, we further studied the combination of IL-6 and IL-10 to identify G-/G+ bacterial infections. Based on the previous ROC curve analysis, the study found that the ratio of IL-6>53.2 pg/ml and IL>24.25 pg/ml was 80.8% in the G- group, while in the G+ group, the ratio of IL-6>53.2 pg/ml and IL ≤ 24.25 pg/ml was 83.7% (**Table 4** and **Figure 2**), and there was a significant difference between the two groups (**Figure 2**, P=0.000). We named the approximately 10-fold increase in IL-6 and the 5-fold increase in IL-10 (IL-6>53.2 pg/ml and IL>24.25 pg/ml) as G- Bacterial Infection Cytokine Profile (G- BICP), and the approximately 10-fold increase in IL-6 and no more than the 5-fold increase in IL-10 (IL-6>53.2 pg/ml and IL ≤ 24.25 pg/ml) as G+ Bacterial Infection Cytokine Profile (G+ BICP). Using G-BICP as the G- Bacterial Infection Cytokine Profile to predict G-/G+ bacterial infection, the specificity was 83.7%, the sensitivity was 80.8%, and the AUC was 0.822 (**Figure 3A**). Using G+ BICP as the G+ B Infection Cytokine Profile to predict G+/G- infection, the specificity was 80.8%, the sensitivity was 83.7%, and the AUC was 0. 822 (**Figure 3B**). Therefore, this study believes that the levels of IL-6 and IL-10 can roughly determine G-/G+ bacterial infection.

**Table 4:** Differentiation of G-/G+ bacterial infections with IL-6 combined with IL-10.

	G-	Blood culture	Overall
		G+	
IL-6>53.2 pg/ml and IL-10>24.25 pg/ml	42	8	49
IL-6>53.2 pg/ml and IL-10 ≤ 24.25 pg/ml	10	41	52
overall	52	49	101

### IL-6/IL-10 Cannot Identify Specific Bacterial Species

We further analysed the IL-6 and IL-10 infected with various bacterial types in group G-. However, no significant difference was observed for the IL-6 and IL-10 among Escherichia coli, Klebsiella pneumoniae, Pseudomonas aeruginosa, Stenotrophomonas maltophilia and other rare bacterial infections (P=0.170,P=0.896, **Table 5**).

**Table 5:** Comparison of IL-6 and IL-10 for various bacteria in the G- group.

	Escherichia coli	Klebsiella pneumoniae	Pseudomonas aeruginosa	Stenotrophomonas maltophilia	Others	P
IL-6	271.1(26.8-5697.9)	639.1(157.3-7553.4)	171.7(70.1-13759.8)	2557.4(1443.0-9937.4)	183.6(59.2-14785.4)	0.170
IL-10	62.2(3.0-2259.1)	40.9(8.0-9327.0)	15(2.6-2848.9)	606.6(18.6-7715.4)	78.3(50.4-1509.4)	0.896

Others: Acinetobacter baumannii and Aeromonas temperate.

### Changes of IL-6 and IL-10 During Treatment

**Figure 4:**
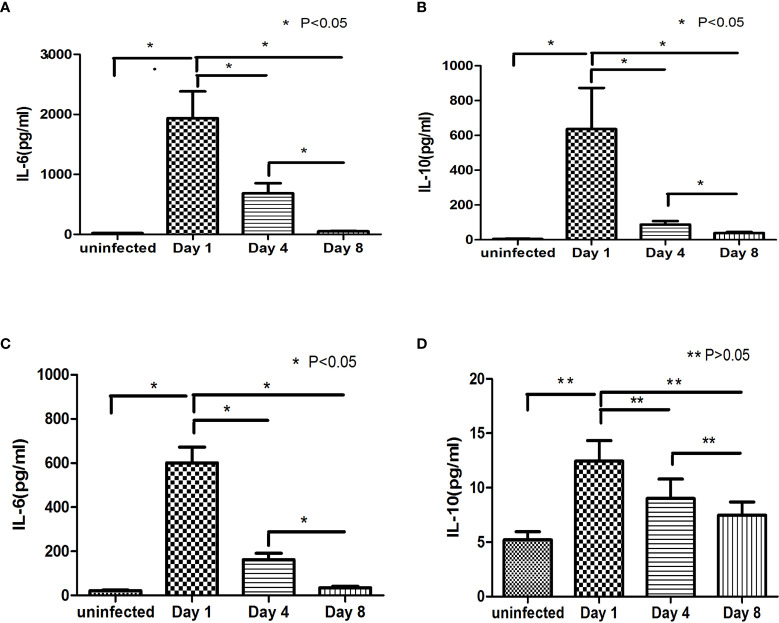
Changes in IL-6 and IL-10 during treatment, **(A**, **B)** show the change trends of IL-6, IL-10 and PCT at day 1, day 4 and day 8 in the G- group and the comparison with the level of the uninfected group. **(C**, **D)** show the change trends of IL-6, IL-10 and PCT in the G+ group at day 1, day 4 and day 8 and the comparison with the level of the uninfected group.

The present study retrospectively analysed the changes in IL-6, IL-10 and PCT in the G- and the G+ groups during treatment (**Table 6** and **Figure 4**). On Day 1, the IL-6, IL-10 and PCT levels of the G- group and G+ group were 386.1(26.8-14785.4) pg/ml, 123.8 (2.6-9327.0) pg/ml, 6.8 (0.4-60.5) ng/ml, and 501.0 (19.3-2134.7) pg/ml, 9.4 (0.5-64.2) pg/ml, and 9.4 (0.5-41.6) ng/ml, respectively. On Day 4, significantly lower IL-6 and PCT levels were observed in both the G- group and the G+ group [89.2 (3.7-5000.5) pg/ml, 2.0 (0.4-13.5) ng/ml; P=0.006, 0.000], [98.3 (2.3-852.8) pg/ml, 1.5 (0.1-8.6) ng/ml; P=0.000, 0.000], respectively. A similar trend was reflected at Day 8, and significantly lower IL-6 and PCT levels were also observed in both the G- group and the G+ group [38.4 (3.6-347.8) pg/ml, 0.6 (0.2-5.0) ng/ml; P=0.000, 0.021, Day 4 vs. Day 8]; [11.9 (1.1-150.7) pg/ml; 0.4 (0.0-1.6) ng/ml; P=0.001, 0.001, Day 4 vs. Day 8], respectively. The IL-10 level of the G- group was significantly decreased at Day 4 [42.6 (1.4-894) pg/ml; P=0.034] and Day 8 [12.3 (0.8-150.7) pg/ml; P=0.039, Day 4 vs. Day 8]. However, there was no significant difference in IL-10 on day 1, day 4 or day 8 in the G+ group (P=0.071). In conclusion, IL-6, IL-10, and PCT decreased gradually on days 1, 4, and 8 in the G- group. Il-6 and PCT decreased gradually on day 1, day 4 and day 8 in the G+ group. In conclusion, as with PCT, we can assess the efficacy of treatment based on changes in IL-6 and IL-10.

**Table 6:** Changes in IL-6, IL-10 and PCT among the four groups.

	G- IL-6 (pg/ml)	G- IL-10 (pg/ml)	G- PCT (ng/ml)		G+ IL-6 (pg/ml)	G+ IL-10 (pg/ml)	G+ PCT (ng/ml)
Day1(A)	52	52	52		49	49	49
	386.1	123.8	6.8	Day 1	501.0	9.4	9.4
	(26.8-14785.4)	(2.6-9327.0)	(0.4-60.5)		(19.3-2134.7)	(0.5-64.2)	(0.5-41.6)
Day4(B)	50^a^	50	50	Day4	46^b^	46	46
	89.2	42.6	2.0		98.3	4.5	1.5
	(3.7-5000.5)	(1.4-894.0)	(0.4-13.5)		(2.3-852.8)	(0.3-68.2)	(0.1-8.6)
Day8(C)	46^c^	46	46	Day8	45^d^	45	45
	38.4 (3.6-347.8)	12.3 (0.8-150.7)	0.6 (0.2-5.0)		11.9 (1.1-150.7)	4. 0(0.3-32.8)	0.4 (0.0-1.6)
P0	0.000	0.000	0.000		0.000	0.071	0.000
P1(A vs. B)	0.006	0.034	0.000		0.000		0.000
P2(A vs. C)	0.000	0.000	0.000		0.000		0.000
P3(B vs. C)	0.000	0.039	0.021		0.001		0.001

P0: P value compares IL-6, IL-10 and PCT among the four groups.

^a^ Data from 2 patients were censored at day 4 of treatment in the G- group

^b^ Data from 3 patients were censored at day 4 of treatment in the G+ group.

^c^ Data from 5 patients were censored at day 8 of treatment in the G- group.

^d^ Data from 5 patients were censored at day 8 of treatment in the G+ group.

## Discussion

Lymphoma is a common haematological malignancy. Chemotherapy is one of the main treatments, but it may damage the immune system and lead to neutropenia, increasing patients’ susceptibility to infection. At present, clinicians often choose antibiotics according to the guidelines on granulocytosis with fever and clinical practice, which may lead to effective efficacy, overuse of antibiotics, emergence of drug-resistant bacteria, and even increase the economic burden of patients. Early and rapid diagnosis of infectious pathogens and timely application of appropriate antibiotics are essential. Early diagnosis and reasonable selection of antibiotics are essential to improve the prognosis of patients and prevent the abuse of antibiotics. The current gold standard for the diagnosis of pathogen types is pathogen culture, and second-generation sequencing can also identify pathogens. However, pathogen culture and second-generation sequencing generally take a long time, which is not conducive to early clinical diagnosis and treatment. The culture of pathogenic bacteria may result in false positives and false negatives. Moreover, second-generation sequencing is more expensive. Commonly used clinical infection markers, such as PCT and CRP, have certain limitations in the diagnosis of infection. The study showed that PCT has poor accuracy in distinguishing bacterial from nonbacterial infections, and PCT may not be an independent marker for the initiation of antibiotic therapy (16). CRP is often elevated in rheumatic connective tissue diseases. PCT and CRP have difficulty distinguishing G- and G+ infections. Therefore, it is urgent to find new and rapid indicators to distinguish G- and G+ infection.

In recent years, there have been some reports on the application of TH1/TH2 cytokines in infectious diseases (5, 11, 14, 17). Many studies have shown that IL-6 and IL-10 are related to infectious disease (18–20). Some scholars even found that IL-6/IL-10 could be used to identify G-/G+ bacterial infections in children (15). IL-6 is one of the newly discovered early markers to assist in the diagnosis of bacterial infections. IL-6 promotes the differentiation of B cells and the secretion of antibody proteins and induces the activation of cytotoxic T cells, thus enhancing immunity (21–23). IL-6 can induce the synthesis of acute-phase reactive proteins such as CRP in acute inflammation. This determination of IL-6 is helpful for the early evaluation and diagnosis of bacterial infection, and its sensitivity and specificity are higher than those of CRP in current clinical use. Its content has a good correlation with the development and prognosis of the disease (24, 25). IL-10 is a pleiotropic cytokine secreted by activated macrophages and T lymphocytes; and it has anti-inflammatory and immunosuppressive effects (22, 23, 26–28). IL-10 prevents the development of immunopathological damage caused by an overprotective immune response in acute and chronic infections. It is also responsible for the persistence of viruses and bacteria by interfering with innate and adaptive immune protection. This study was based on the detection of TH1/TH2 cytokines in peripheral blood based on flow cytometry (FCM). This technique has the advantages of high throughput, rapidity, and good reproducibility and is suitable for clinical analysis. With the progress of flow cytometry technology, CBA technology provides a powerful multichannel detection technology. Compared with the traditional enzyme-linked immunoadsorption assay (ELISA), the CBA method can quickly detect multiple cytokines, using lower sample sizes, labour costs and time consumption.

In this study, we found that there was no significant difference in IL-2 and IL-4 among the healthy, uninfected and infected groups. The INF-γ and TNF-α levels of lymphoma patients were higher than those of healthy controls. This may be because IFN-r and TNF-α are closely related to the occurrence and development of lymphoma, which is consistent with the results of a previous study (29, 30). We found that compared with healthy people and uninfected lymphoma patients, IL-6 and IL-10 were significantly increased in G- bacterial infections, while IL-6 was significantly increased and IL-10 was not significantly increased in G+ bacterial infections. The ROC value showed that IL-10 could distinguish between patients in the G- and G+ groups (**Figure 1A**, AUC = 0.904, P = 0.000). The cut-off value of IL-10 was 24.25 pg/mL, and its sensitivity and specificity were 87.8% and 84.6%, respectively. IL-6 could distinguish the infected group from the uninfected group (**Figure 1B**, AUC=0.990, P=0.000), the cut-off value of IL-6 was 53.20 pg/mL, and the sensitivity and specificity were 96.0% and 94.1%, respectively. However, since IL-6 showed no significant difference between the G- and G+ groups, it is obviously difficult for a single cytokine to distinguish G- and G+ bacterial infections in all populations. Combining the aforementioned ROC critical value, we named the approximately 10-fold increase in IL-6 and the 5-fold increase in IL-10 (IL-6>53.2 pg/ml and IL>24.25 pg/ml) as G- Bacterial Infection Cytokine Profile(G- BICP), and the approximately 10-fold increase in IL-6 and no more than the 5-fold increase in IL-10 (IL-6>53.2 pg/ml and IL ≤ 24.25 pg/ml) as G+ Bacterial Infection Cytokine Profile (G+ BICP). Tang et al. found that in childhood haematological tumours, IL-6 and IL-10 both increased by more than 10 times in G- bacterial infection, but the increase of the IL-10 was less than 10 times in G+ bacteria bacterial infection (15). This is consistent with the general direction of our research, but the value of IL-10 is slightly different, which may be related to the patient’s age and bone marrow suppression status after chemotherapy. Guan found that IL-6 and IL-10 were closely related to bloodstream infection and suggested that IL-10 might distinguish G- and G+ infection, but the diagnostic effect of IL-10 was not ideal, with a sensitivity of 64.1%. The specificity was 70.9% (31). Guan’s study did not observe the significance of the combination of IL-6 and IL-10 for the differentiation of G- and G+ infection, which may be caused by factors such as the different types of diseases of the study subjects (31). In this study, for the first time, we proposed the combination of IL-6 and IL-10 to identify G-/G+ bacterial infections in adult lymphoma during the myelosuppression stage after chemotherapy. The specificity and sensitivity of using IL-6 combined with the IL-10 cytokine to identify G- and G+ bacterial infections were both greater than 80%.

In addition, the present study found that PCT and IL-6 decreased gradually at day 4 and day 8 of treatment in the G- and G+ groups. A similar trend was reflected in the IL-10 level of the G- group. Some studies have shown that PCT can be used as an indicator of infectious diseases, infection severity and disease prognosis (32–34). However, a single PCT has some limitations, and other indicators often need to be combined in clinical practice (35). The present study suggested that dynamic monitoring of IL-6 and IL-10 can be used to evaluate the efficacy of anti-infective therapy, which is consistent with Guan’s research results (31). More observation time points were observed in this study for the changes in IL-6 and IL-10. Clinicians can preliminarily judge whether patients are infected and whether the infections are caused by G- or G+ bacteria; according to the cytokines, they can adjust antibiotic treatments in a timely manner and evaluate the effect of anti-infection treatment through dynamic monitoring of IL-6 and IL-10 changes.

In conclusion, this study suggests that cytokines, especially IL-6 and IL-10, can preliminarily identify lymphoma G-/G+ infections, which is conducive to early, timely and targeted selection of antibiotics. Il-6 and IL-10 can be used as indicators of anti-infection treatment effects. As a single-centre retrospective study, the present study has several limitations. The accuracy of IL-6/IL-10 in determining G-/G+ was less than 100%, and specific strains such as Escherichia coli or Klebsiella pneumoniae could not be identified. Although these results did not attain statistical significance, We observed that the IL-6 and IL-10 levels of the patients infected with Stenotrophomonas maltophilia were markedly higher than for the other groups of gram-negative bacteria. There were only 4 cases of stenotrophomonas maltophilia patients in our study. A larger sample size is needed in the future to investigate whether IL-6 and IL-10 levels are higher in patients infected with stenotrophomonas maltophilia than the patients infected with other gram-negative bacteria. In clinical practice, antibiotics should be selected according to cytokines, clinical symptoms, experience and guidelines.

## Data Availability Statement

The original contributions presented in the study are included in the article/supplementary material. Further inquiries can be directed to the corresponding authors.

## Ethics Statement

The studies involving human participants were reviewed and approved by the Ethics Committee of the First Affiliated Hospital of Guangdong Pharmaceutical University. Written informed consent for participation was not required for this study in accordance with the national legislation and the institutional requirements.

## Author Contributions

QZ and HL conceptualized and designed the study. QZ, HL, and SZ collected and analyzed data. QZ drafted the paper. QZ, BW, ML, and XP carried out the data analysis, and revised the paper. All authors contributed to manuscript review, read, and approved the submitted version.

## Funding

This study was supported by the Science and Technology Project of Yuexiu District of Guangzhou (2017-W S-008), the Natural Science Foundation of Guangdong Province (2017A030313664), and the Science and Technology Planning Project of Guangzhou (202002030253).

## Conflict of Interest

The authors declare that the research was conducted in the absence of any commercial or financial relationships that could be construed as a potential conflict of interest.

## Publisher’s Note

All claims expressed in this article are solely those of the authors and do not necessarily represent those of their affiliated organizations, or those of the publisher, the editors and the reviewers. Any product that may be evaluated in this article, or claim that may be made by its manufacturer, is not guaranteed or endorsed by the publisher.
